# Bacterial Community Composition of South China Sea Sediments through Pyrosequencing-Based Analysis of 16S rRNA Genes

**DOI:** 10.1371/journal.pone.0078501

**Published:** 2013-10-21

**Authors:** Daochen Zhu, Shoko-Hosoi Tanabe, Chong Yang, Weimin Zhang, Jianzhong Sun

**Affiliations:** 1 School of Environmental Engineering, Jiangsu University, Zhenjiang, Jiangsu, China; 2 State Key Laboratory of Applied Microbiology in South China (Ministry-Guangdong Province Jointly Breeding Base), Guangdong Institute of Microbiology, Guangzhou, China; 3 School of Environmental Science, University of Shiga Prefecture, Hikone, Shiga, Japan; 4 Department of R&D, Zhenjiang Bio-innova Biotech Co, Ltd., Zhenjiang, Jiangsu, China; Wageningen University, Netherlands

## Abstract

**Background:**

Subseafloor sediments accumulate large amounts of organic and inorganic materials that contain a highly diverse microbial ecosystem. The aim of this study was to survey the bacterial community of subseafloor sediments from the South China Sea.

**Methodology/Principal Findings:**

Pyrosequencing of over 265,000 amplicons of the V3 hypervariable region of the 16S ribosomal RNA gene was performed on 16 sediment samples collected from multiple locations in the northern region of the South China Sea from depths ranging from 35 to 4000 m. A total of 9,726 operational taxonomic units (OTUs; between 695 and 2819 unique OTUs per sample) at 97% sequence similarity level were generated. In total, 40 bacterial phyla including 22 formally described phyla and 18 candidate phyla, with *Proteobacteria, Firmicutes, Planctomycetes, Actinobacteria* and *Chloroflexi* being most diverse, were identified. The most abundant phylotype, accounting for 42.6% of all sequences, belonged to *Gammaproteobacteria*, which possessed absolute predominance in the samples analyzed. Among the 18 candidate phyla, 12 were found for the first time in the South China Sea.

**Conclusions:**

This study provided a novel insight into the composition of bacterial communities of the South China Sea subseafloor. Furthermore, abundances and community similarity analysis showed that the compositions of the bacterial communities are very similar at phylum level at different depths from 35-4000 m.

## Introduction

Advances in high-throughput sequencing, also known as next-generation sequencing technology, including 454 pyrosequencing, Illumina sequencing etc., has significantly promoted microbial diversity and ecological studies. Deep sequencing makes it possible to precisely describe complicated microbial communities in several environments including marine, soil, animal or insects guts, which were all over 100 times more diverse than previously reported by traditional culture-dependent methods [1–4]. Marine microbial communities mediate biogeochemical ocean cycles including carbon, nitrogen and sulphur, and are probably play pivotal roles in maintaining marine ecosystem to prevent environmental changes such as warming and ocean acidification [5,6]. The ocean microbial community structure is influenced by pH, water temperature, salinity, silicate, seasonal shifts, and ocean currents as proved by surveys of the La Sal del Rey hypersaline lake located in southern Texas, USA [7], the western English Channel [8], and the western Arctic Ocean [9]. Phylotypic richness differed between summer and winter, but remarkable bacterial community structure stability was observed over time in the western Arctic Ocean [9]. Previously, the abundance of phylotypes in the oceanic microbial community was focused on abundant species because they were easily detectable. Thereafter, the 454 pyrosequencing technique revealed that most of the diversity of oceanic microbial communities is comprised of a high number of rare species and, in some cases, collectively comprise up to 75% of the abundance in their communities, named ‘‘the rare biosphere” [3,9,10]. The rare microbial biosphere together with abundant members play important roles in the functioning of the ocean ecosystem [11]. 

The South China Sea is one of the largest marginal seas and lies within the West Pacific marine. The detrital fluxes of sediments of South China Sea came from three of the largest rivers in the world (Mekong River, Red and Pearl rivers), and the monsoon activity plays an important role in the cycling of organic carbon and other biogenic  component of sediments, which controlled the sea surface circulation. In summer, the subtropical waters are advected into the South China Sea through the southern straits and through the Taiwan Strait to exit the South China Sea. In winter, the cold and saline waters enter the South China Sea from the north as a reversed pattern [12].

It has an average water depth of 1200 m and a maximal depth of approximately 5380 m and has long been recognized as the global center of marine tropical biodiversity [13]. There are abundant organic matters in the deposited sediments of the seafloor [14]. Bacterial community richness estimated from rRNA sequences of ocean samples revealed hundreds to thousands of phylotypes [15,16]. To date, the South China Sea bacterial community distribution patterns remain unknown. Although a few surveys based on culture-dependent, denaturing gradient gel electrophoresis and constructed PCR product clone libraries methods analyzed the bacterial diversity, the obtained numbers of operational taxonomic units (OTUs) was lower than100 sequences for each sample [17–22]. These smaller datasets result in the underestimation of species richness and generally do not describe rare populations that might represent considerable diversity.

The goal of our study was to explore questions about bacterial diversity in subseafloor sediments of the South China Sea using sequences of the V3 region of the 16S rRNA gene as determined by 454 pyrosequencing. We examined 16 sediment samples obtained from the surface of shallow and deep-sea bottoms at depths from 35 to 4000 meters. We next compared the similarity of rare and abundant phylotypes of the communities. Furthermore, we described the taxonomic composition of bacterial communities from our samples.

## Results and Discussion

To investigate bacterial diversity and provide an in-depth description of relative abundance in benthic regions of the South China Sea, 454 (Life Science, Branford, CT, USA) pyrosequencing technology was used to sequence 16 samples collected from different locations of the northern region of the South China Sea, with depths ranging from 35-4000 m (Table 1). We sequenced more than 265,000 PCR amplicons that span the V3 hypervariable region of rRNAs from our DNA preparations. Each sample produced from 13,000 to 20,000 reads. To eliminate random sequencing errors, reads were trimmed by removing the barcode and primers. Sequences with lengths less than 150bp or with ambiguous residues were also discarded. After removing potential erroneous sequences, data for the 16 samples was reduced from 21.8% to 10%, and on average data sizes were reduced by12.4%. 

**Table 1:** Environmental DNA samples used for sequence analyses from the South China Sea.

Sample ID	Lat °N, Long °W	Date	Depth, m
1	111°23.243', 17°59.925'	28/08/2011	1937.5
2	111°29.456', 17°58.927'	28/08/2011	2026.6
8	112°08.124', 18°0.541'	29/08/2011	2448.3
11	114°30.315', 18°1.841'	31/08/2011	3563
12	117°2.913', 18°1.742'	01/09/2011	3938
13	119°31.949', 18°2.114'	02/09/2011	3023
15	119°44.263', 18°44.606'	02/09/2011	3415
16	120°0.250', 20°22.971'	04/09/2011	3536
17	119°19.896', 19°41.569'	05/09/2011	2918
18	117°37.208', 21°23.202'	06/09/2011	652
19	116°30.202', 22°29.355'	06/09/2011	35
20	116°22.842', 20°38.176'	07/09/2011	431
21	116°43.681', 20°14.109'	07/09/2011	717
22	117°58.233', 19°0.368'	08/09/2011	3739
25	115°17.868', 19°42.407'	09/09/2011	1808
27	109°28.614', 18°13.299'	26/08/2011	300

Lat, latitude, Long, longitude

### Taxonomic richness of benthic bacterial communities of South China Sea

Based on a BLASTN search of trimmed 454 reads in the RefHVR_V3 database to identify the closest matches, sequence tags were clustered into groups by defining the variation from unique sequences to 10% differences. These clusters were calculated for OTUs, abundance-based coverage estimator (ACE), and the Chao1 estimator. The exponential Shannon index was calculated and the Simpson index at species, genus, and family levels were defined with the sequence similarity thresholds of 97%, 95%, and 90%, respectively. Rarefaction curves were generated based on a species level. In total, 9,726 unique OTUs at the 97% threshold were obtained from the 16 samples. 

**Figure 1:**
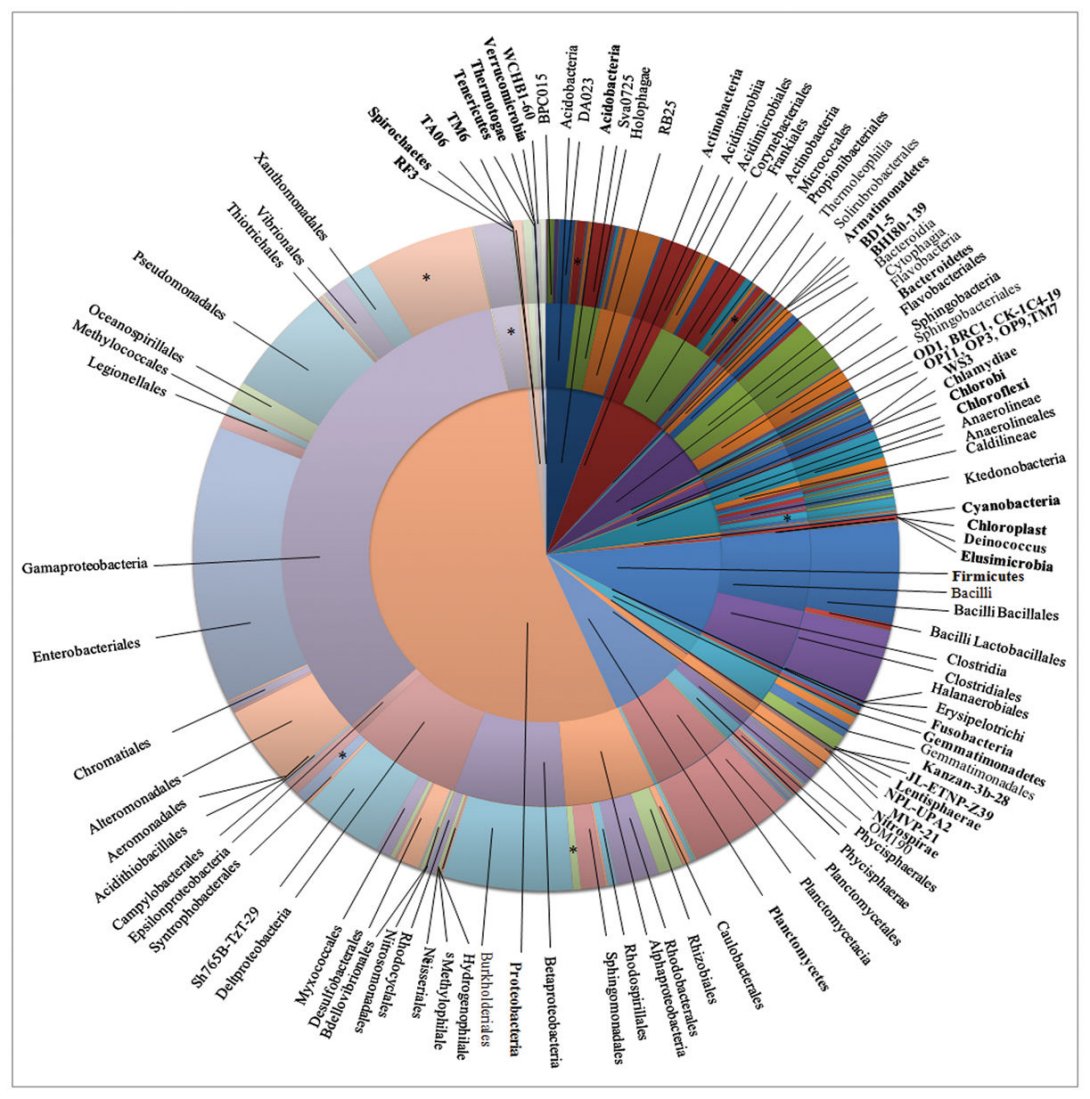
Bacterial diversity richness and phylogenetic distribution on phylum (inner circle), class (middle circle), and order level (outer circle) (Based on 97% OTUs). The shown phyla include formally described taxonomically phyla and candidate phyla. Phylum names are given in bold. Selected class and order groups are labeled. *represents unclassified groups.

**Figure 2:**
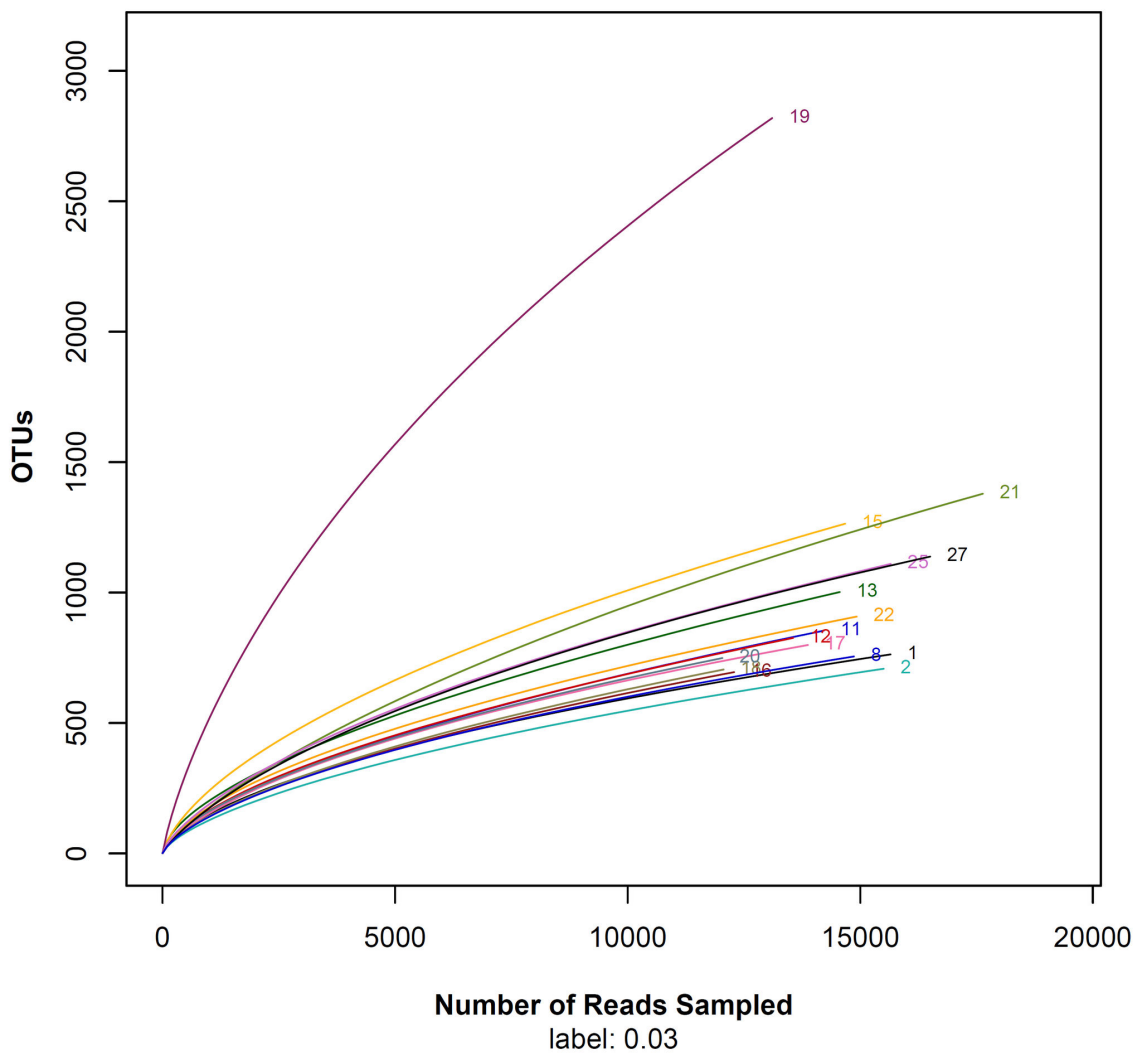
Rarefaction analysis for the 16 sediment samples. The curves were generated for 97% levels of OTU.

A ranges of 695 (sample 16) to 2819 OTUs (sample 19) were discovered in a total of 16 benthic sediment samples (Table 2). At the phylum level, all OTUs could be classified and belonged to 22 formally described bacterial phyla and 18 candidate phyla (Figure 1). Therefore, the overall known diversity in the South China Sea increases to 40 different bacterial phyla and candidate phyla, which was higher than the reported number of 35 phyla from other marine habitats, including the Arctic Ocean and the Western English Channel [8,11]. In this study, we used massively parallel signature sequencing technologies to obtain more than 265,000 sequences from 16 sediment samples from depths of 35 to 4000 m in a 1 million square kilometer area surrounded by the Sanyan Bay, Luzon Island, Shantou bay, and Paracel Islands to the west, north, east, and south. The rarefaction analysis of the OTUs indicated that the bacterial community diversity of sample 19 (depth <300 m) was significantly higher than other samples (depth >300 m; Figure 2). In addition, the similar result also was obtained from other sea area samples. For example, in a study to investigate the prokaryote diversity in the sub-seafloor biosphere (Accession: SRP001218), 79717 OTUs (97% genetic similarity) were obtained from three shallow sea sediments (~3.5 m) but only 46836 OTUs were obtained from three deep sea samples (depth 3860 m and 1326m) (Figure S1A). In another study (SRP001269) for investigating the microbial biodiversity of Indian Ocean region revealed the average amount of OTUs was 25367 for the shallow sea sediment samples, and the average amount of deep sea samples OTUs only reached 17556. This suggests that the bacterial community diversity of the shallow subseafloor near the coast was richer than the deep subseafloor area. 

**Table 2:** Similarity-based OTUs and species richness estimates.

		Cluster distance														
		0.03					0.05					0.10				
Sample ID	reads	OUT	ACE	Chao	Shannon	Simpson	OUT	ACE	Chao	Shannon	Simpson	OUT	ACE	Chao	Shannon	Simpson
1	15715	763	2403	1601	2.73	0.3279	527	1221	910	2.51	0.3586	277	381	396	2.21	0.3924
2	15536	708	2310	1526	2.33	0.4004	487	1130	837	2.14	0.4202	247	341	344	1.81	0.4562
8	14926	755	2400	1646	2.37	0.4047	533	1175	935	2.24	0.4078	271	341	337	1.96	0.4423
11	14290	854	3505	2081	3.59	0.1024	623	1734	1134	3.4	0.108	357	606	480	3.18	0.1158
12	13604	826	3087	1959	3.04	0.2487	596	1501	1198	2.83	0.2671	329	454	483	2.55	0.2916
13	14623	1002	4176	2475	4.16	0.0783	718	2252	1408	3.9	0.0855	416	832	650	3.55	0.095
15	14783	1264	4422	2872	4.32	0.061	950	2550	1807	4.01	0.0825	568	851	834	3.69	0.0966
16	12358	695	2597	1559	2.46	0.3775	485	1221	892	2.31	0.3801	242	330	323	1.97	0.4219
17	13978	799	2397	1717	2.52	0.3879	585	1339	1055	2.37	0.3947	349	483	481	2.08	0.4304
18	12139	705	2512	1768	2.59	0.3473	512	1293	1070	2.46	0.3501	268	366	355	2.16	0.3848
19	13287	2819	8559	5950	6.52	0.0056	2207	5358	4123	6.25	0.0067	1250	2201	1958	5.68	0.0088
20	12112	749	2446	1716	2.96	0.2785	530	1187	899	2.79	0.2884	293	383	372	2.52	0.3127
21	17768	1379	5367	3165	3.47	0.0976	1039	3151	2225	3.14	0.1234	642	1526	1188	2.85	0.1333
22	14999	908	3282	2139	3.03	0.283	645	1757	1202	2.88	0.2869	349	624	543	2.59	0.313
25	15750	1110	3782	2612	3.7	0.1163	839	2150	1538	3.51	0.1204	513	870	719	3.24	0.1311
27	16572	1138	3430	2506	2.52	0.407	843	2019	1561	2.38	0.4094	468	640	631	2.02	0.4532

**Figure 3:**
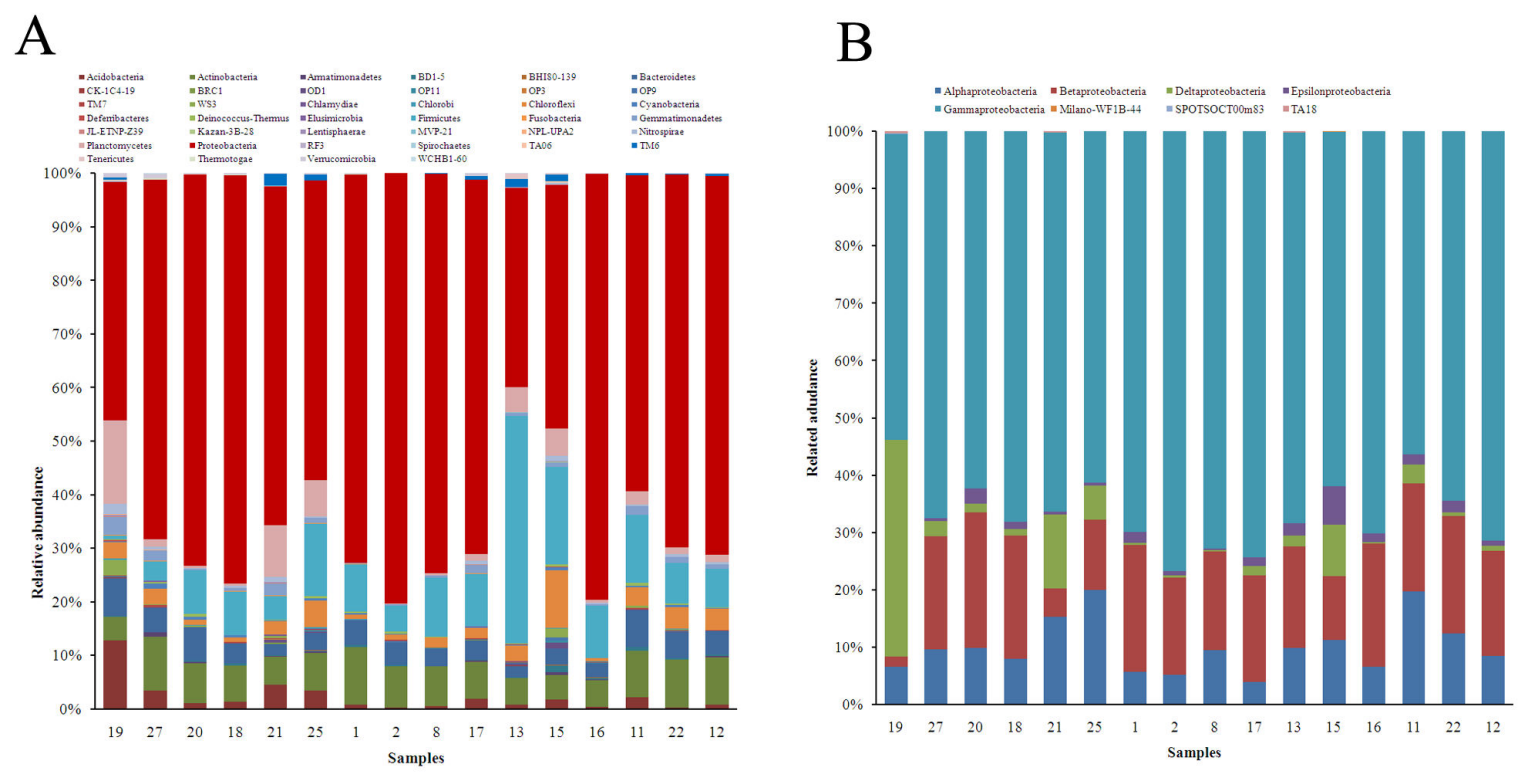
Bacterial community distribution in 16 sediment samples. A, the relative abundances of different phyla; B, the relative abundances of different classes in Proteobacteria. The relative abundance is presented in terms of percentage in total effective bacterial sequences per sample.

Abundance analysis showed that nine phyla account for over 95% of the total amplicons. The phyla include *Proteobacteria, Firmicutes, Planctomycetes, Acidobacteria, Actinobacteria, Chloroflexi, Bacteroidetes, Gemmatimonadetes*, and *Nitrospirae* (Figure 3A). *Proteobacteria* was the most abundant phylum in all samples and accounted for 37-80% of all bacterial amplicons. As the most dominant community in marine environments, *Proteobacteria* has also been described in the Arctic Ocean [11], marine sponges [1], and the benthic North Sea [23]. 

The dominant communities of sample 19 were *Proteobacteria* (44.7%), *Planctomycetes* (15.5%)*, Acidobacteria* (12.9%)*, Bacteroidetes* (7.0%), and *Actinobacteria* (4.5%). In the remaining samples, *Proteobacteria*, *Firmicutes, Actinobacteria, Chloroflexi* and *Bacteroidetes* accounted for 11-42% of effective sequences. This suggests that in shallow sea sediments, the phyla *Planctomycetes* and *Acidobacteria* are the second and third dominant communities, accounting for 28.3% of tags. However, in deep-sea sediments, *Firmicutes* and *Actinobacteria* are the second and third most dominant communities, representing 8.9% to 45.2% of the tags. *Planctomycetes* and *Acidobacteria* only accounted for 0.4% to 14.8% of tags from overall deep sea sediment samples. 

In *Proteobacteria, Gammaproteobacteria* were the most dominant class in all samples, accounting for 53.4% to 76.8%, and *Deltaproteobacteria* was the second most dominant class, accounting for 37.8% of tags in sample 19. However, *Alphaproteobacteria* and *Betaproteobacteria* were the second and the third most dominant classes in the deep-sea sediment samples (Figure 3B). *Gammaproteobacteria*, the predominant bacterial group, prevailed over other taxa identified in several deep-sea investigations, including the Eastern Mediterranean Sea [24] and Northeastern Pacific Ocean [25]. Sequences affiliated with *Desulfobacterales*, *Myxococcales*, and Sh765B-TzT-29, dominated the *Deltaproteobacteria* and their common role is to regulate the sulfur cycle. The ocean represents a major reservoir of sulfur on Earth and microbial transformation of sulfur compounds has had a profound effect on the properties of the biosphere and continues to affect geochemistry [26]. The types of sulfur-metabolizing microorganisms of *Deltaproteobacteria* include sulfate reducers, organic sulfur utilizers, and sulfur reducers [27].

Abundance analysis of bacterial community diversity comparing the South China Sea with other marine area sediment samples was performed at the phylum level and the class level for *Proteobacteria*. The bacterial communities of different marine area displayed similarity in dominant groups, which including *Proteobacteria* (*Gammaproteobacteria*, *Deltaproteobacteria*)*, Planctomycetes, Firmicutes, Actinobacteria, Acidobacteria*, *Bacteroidetes*, and *Chloroflexi* (Figure S1). The phylum *Proteobacteria*, being most dominant group, was observed in a large proportion of shallow sea and deep-sea sediments. In the global overview, the dominant groups of bacterial communities of shallow sea sediments were similar with that of deep-sea sediments in the same sea area. 

The benthic bacterial communities of the South China Sea showed similarity with other sea areas, but each area still has different characteristics in their dominant group’s proportions. Such as in samples from cluster CFU1 (Atlantic Ocean near Portugal), where more than 54.7% of the sequences belong to candidate phylum OP9; in a 5000 m depth benthic sample from the Indian Ocean, the class *Alphaproteobacteria* reached 93.4% in total sequences. The geographical location has a strong impact on microbial community composition, and explained 22.2% of the observed differences in benthic communities[16]. 

### Principal-component analysis of the bacterial community of the South China Sea

**Figure 4:**
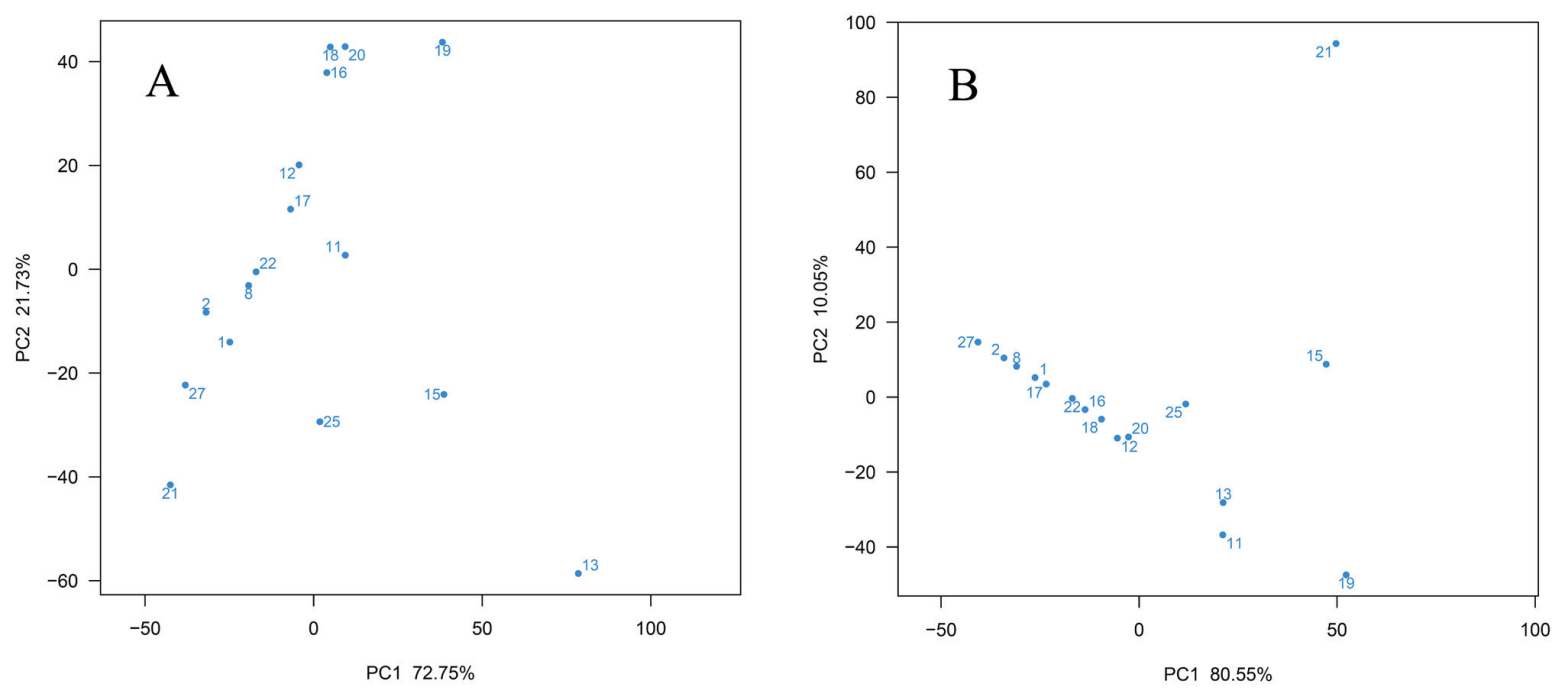
Principal-component analysis of bacterial communities in the South China Sea. A, in the level phylum; B, in the level OTU 0.03. The Number refers to the samples ID which is explained in Table 1.

To determine the distribution and biogeography of the bacterial community, the 454 data were analyzed in relation to sampling locations using principal-component analysis (Figure 4). The similarity of microbial communities among our 16 samples, collected from depths of 35-4000 m, was monitored with PCA at the phylum level and OTU0.03 levels. At the phylum level, samples collected from similar depths or locations did not contain more similar microbial communities to one another than to samples collected at other depths or locations (Figure 4A). For example, samples 16, 18, and 20, collected from depths of 3536 m, 652 m and 431 m, respectively, fell into a cluster, while samples 19, 20, and 21 were all collected from the Shantou bay but fell into different clusters. However, a large amount of deep-sea samples clustered into a group by PCA analysis at the OTU 0.03 levels (Figure 4B). The deep-sea samples exhibited a noticeable and regular separation from shallow sea in the first principal component (PC1), and 80.55% of the variation in the data explained in PC1. A large part of deep-sea samples corresponded to negative values and the shallow sea sample 19 had the highest value, which was more than 50. The shallow sea sample 19 had the lowest negative value in the second principal component (PC2), which represented 10.05% of the variation; a large amount of deep-sea samples fall in a range from -20 to +20. 

In addition, the richness of bacterial diversity of the deep subseafloor was less than that of the shallow subseafloor (Figure 2). Because the nutrient-limited, low energy-flux, and high press environment of deep subseafloor leads to microbial abundance, activity and turnover rates in the deep subseafloor are extremely low relative to those in other global habitats [28]. 

### Rare biosphere in South China Sea

Deep sequencing revealed that the rare biosphere accounts for tremendous diversity of marine bacterial communities. We defined the rare phylotypes as having a frequency <0.01% and abundant phylotypes at a frequency >1% within a sample, according to previous reports [11,29] .

**Figure 5:**
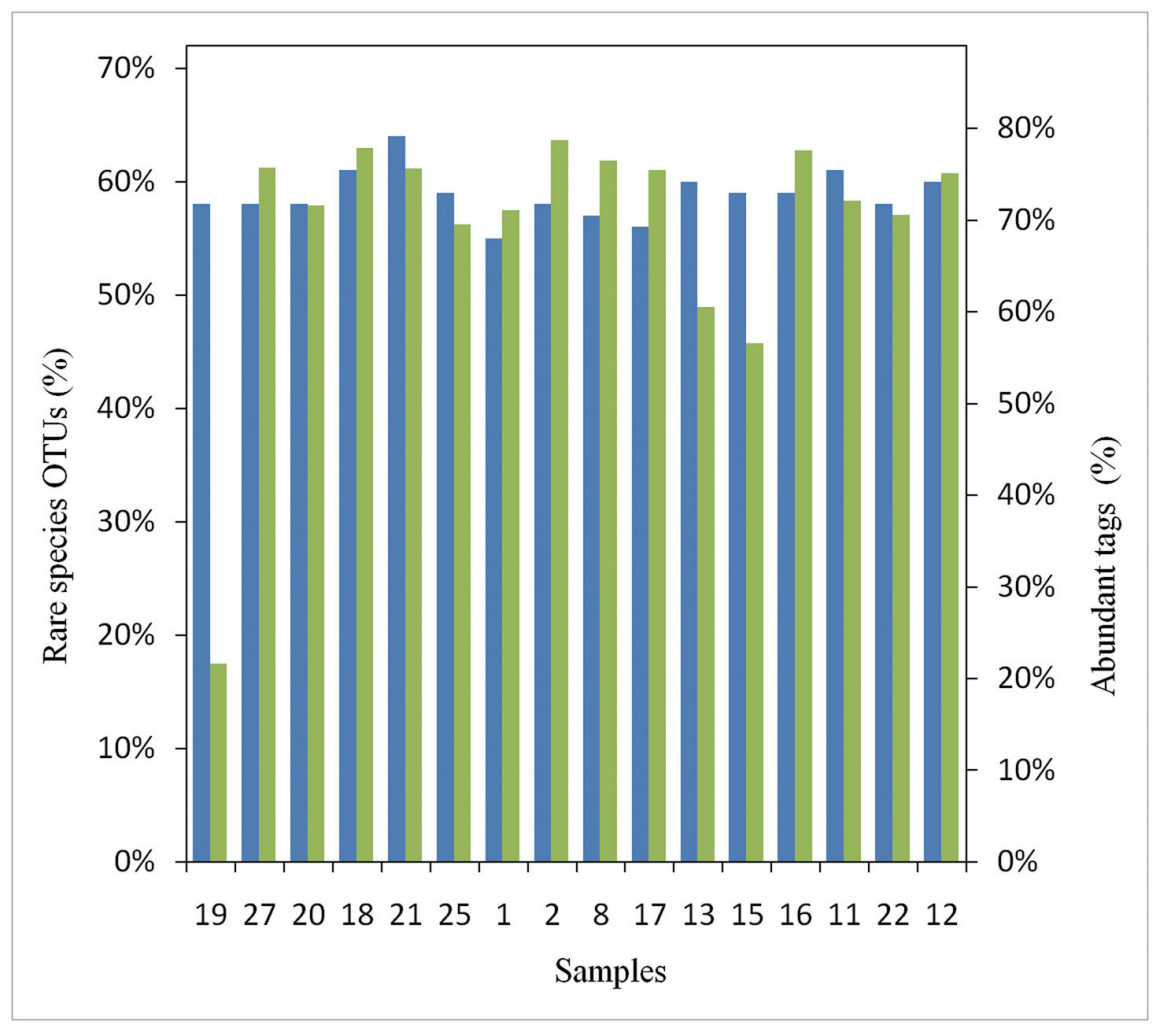
Phylogenetic composition of rare bacterial phylotypes (≤0.01%) and richness of abundant phylotypes (≥1%frequency) for the 16 sediment samples.

The rare phylotype distribution in each sample was similar and within a range of 55-64% of OTUs but comprised <5% of the sequence abundance (Figure 5). Overall, 62 abundant phylotypes were counted in shallow sea and deep-sea sediment samples, covering 57-76% and 22% of the number of sequences, respectively. However, this only comprised <1.5% of the OTUs. In addition, 18 phylotypes of 62 abundant phylotypes were found to be abundant in some samples but rare in others. For example, the hydrocarbon-degrading *Gammaproteobacteria*, *Cycloclasticus*, a member of the rare biosphere, becomes an abundant member of the community when supplied appropriate conditions but returns to the rare biosphere by the loss of the supplied condition [30]. This suggests that the distribution of the bacterial rare biosphere is not obviously different in shallow and deep-sea sediments. The most abundant phylotype belonged to *Gammaproteobacteria*, accounting for 42.6% of total sequences and possessing absolute predominance in the South China Sea sediments. Interestingly, with the exception of the most abundant phylotype, 13 of 14 abundant phylotypes from sample 16 were rare or absent in other samples. Probably the difference of the sediments environment of deep and shallow sea such as temperature, pH, pressure, enrichment in reduce of compounds, and metals bring the variation of abundant phylotypes. In addition, the seasonal cycle brings environmental changes from the Pacific coast, which results in these populations dominating the assemblage during a period of time then declining in the abundant biosphere to the point where they become undetectable [31]. The majority of sequences from the community were occupied by abundant taxonomic groups. These groups were thought to be well adapted to the environment and to contribute the most to biomass production [32,33]. Conversely, the biomass of rare groups is negligible compared to that of the abundant members of the community, and their contribution to carbon flow is relatively small. However, some members of the rare biosphere that are actively growing can significantly contribute to particular elements such as nitrogen and sulfur cycling. For example, *Desulfosporosinus* account for <0.01% of the total cell count but could contribute to most of the sulfate reduction in peat [34,35]. 

Further analyses of community structure and function are needed to investigate the interaction between rare phylotypes and marine subseafloor habitats. The 454 pyrosequencing technique still has pitfalls and may affect our results for the rare biosphere. Even the largest published metagenomic investigations inadequately represent the full extent of microbial diversity, and primer efficiency for generating 16S rRNA gene fragments is limited [10,36].

## Conclusions

Overall, this study is the first metagenomic analysis using pyrosequencing to characterize a more comprehensive overview of the bacterial community of the South China Sea. The massively parallel signature sequencing of 16 samples of subseafloor sediments and data analysis allowed novel insights into the complex composition of this microbial community. Detected diversity of bacterial communities increased to 40 different bacterial phyla and 18 candidate phyla, and the majority populations of the South China Sea at different depths from 35-4000 m showed a high similarity at the phyla level. However, the shallow subseafloor showed a higher bacterial diversity compared to deep subseafloor. 

## Materials and Methods

### Sample collection and preparation for pyrosequencing

**Figure 6:**
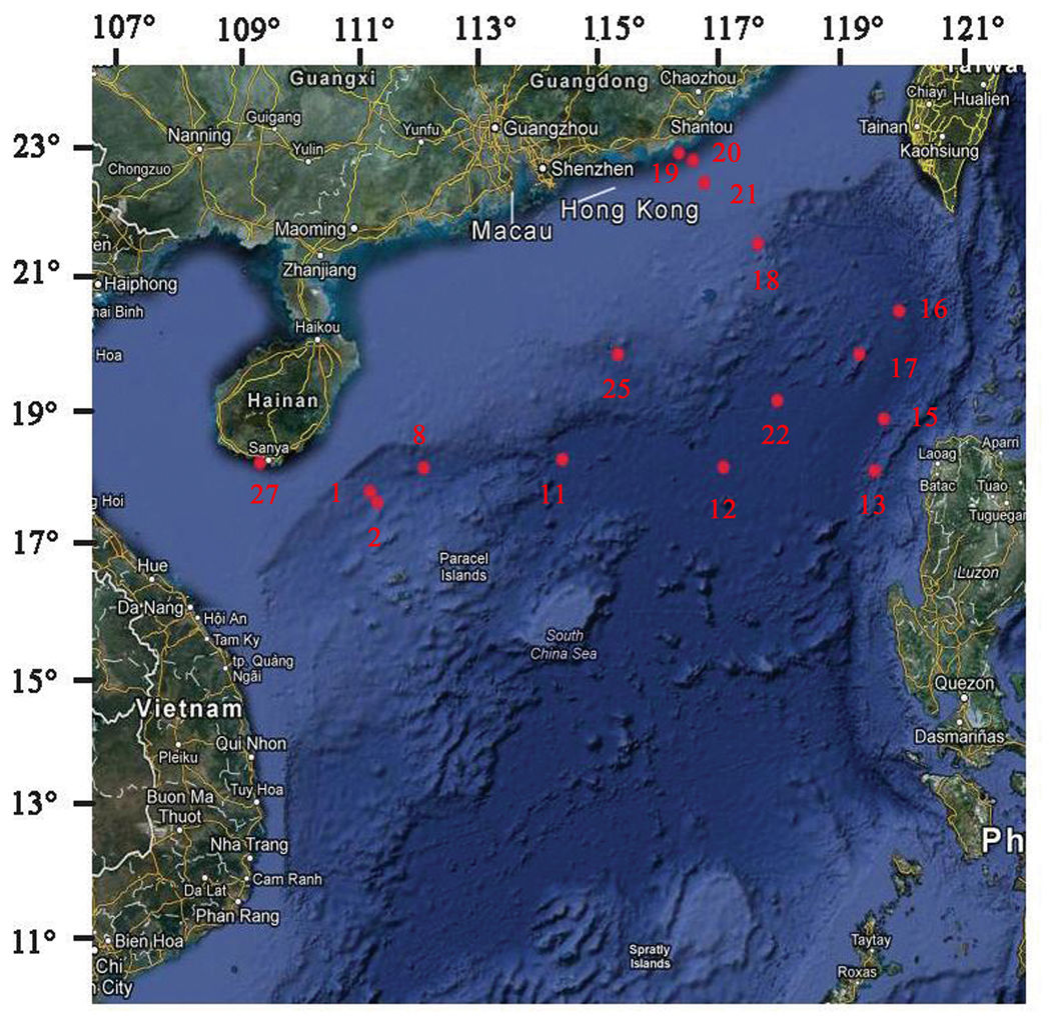
Map of sampling locations in the South China Sea.

Samples were collected from the deposited sediment of benthic regions in 16 different locations of the northern region of South China Sea (between Lat °N, 111° 23.243' and 120° 0.250' to Long °W 17° 58.927' and 22° 29.355') at water depths ranging from 35-4000 m. Samples were transferred to sterilized plastic tubes and stored at -80°C (Figure 6). There are no specific permits required for the described sampling because collections did not involve endangered species and did not occur within a designated marine protected area, private reserve, or park.

Total genomic DNA was extracted from 1g of the sediment samples using the EZgeneTM Soil gDNA Kit (Biomiga, San Diego, CA, USA) according to the manufacturer’s protocols. Bacterial 16S rRNA at the V3 hypervariable region were amplified using a set of primers designed by adding a 10-nucleotide barcode to the forward primer, 8F, (5’-AGAGTTTGATCCTGGCTCAG-3’) and reverse primer, 533R, (5’-TTACCGCGGCTGCTGGCAC-3’). The amplification reaction mixture contained 5 Units of Pfu Turbo DNA polymerase (Stratagene, La Jolla, CA, USA), 1×Pfu reaction buffer, 200 µM dNTPs (TaKaRa, Dalian, China), 0.2µM barcoded primer, and 20ng genomic DNA template for a total volume of 100 µl. PCR was performed with a thermal cycler (Bio-Rad, USA) under the following condition: 5 min at 94°C, 25 cycles of 30 s at 94°C plus 45 s at 55°C plus 30 s at 72°C, and finally 5 min at 72°C. The PCR products were purified by using a PCR Purification Kit (QIAGEN, Hilden, Germany). The 200 ng purified amplicons from each sample were mixed then pyrosequenced on the ROCHE 454 FLX Titanium platform (Roche, Basel, Switzerland) at Majorbio Bio Tech Co. Ltd (Shanghai, China). The results of the raw data have been deposited into the EMBL/GenBank/DDBJ Nucleotide Sequence Data Libraries under the Accession Number: DRA000705.

### Sequence analysis

Raw sequence reads were filtered to eliminate the effect of random sequencing errors. The primer and barcode of each read were removed and trimmed. The sequences that (i) were shorter than 150 nucleotides, (ii) contained ambiguous bases (N), or (iii) contained homopolymer regions (>6 repetitions of the same base) were excluded. 

Taxonomic identification of the reads (‘‘tags’’) were performed following the process described by Sogin et al [3]. All optimized reads were trimmed down to equal lengths (150bp), which contained the V3 hypervariable region. The tags were searched using the NCBI BLASTN tool in the reference database of hypervariable region tags (RefHVR_V3, http://vamps.mbl.edu/) based on the SILVA database, version 106 [37], and queried reads showing the minimum distance with the reference V3 tags were grouped and assigned the same phylotype. Taxonomy was assigned to each trimmed reference sequence (400bp) with Mothur 1.24.0 [38].

The reference ICoMM 454 bacterial 16S pyrotag dataset for comparing with data of this study were obtained from the internet (VAMPS website: http://vamps.mbl.edu, MICROBIS website: http://icomm.mbl.edu/microbis) and are provided in Table S1; sample descriptions were from Zinger, L. et al. [15].

### Diversity and statistical analysis

The sequences were grouped into OTUs sharing ≥97%, ≥95%, or ≥90% similarity by DOTUR [39]. The bacterial community richness indices (non-parametric ACE and the Chao1) and diversity indices (Shannon and Simpson estimators) were calculated using Mothur and Shannon-ace-table.pl software programs (Majorbio, Shanghai, China). Rarefaction curves were calculated using Mothur and the software program Plot-rarefaction (Majorbio, Shanghai, China). Heat maps were drawn by hierarchal clustering performed in the R software environment (http://www.R-project.org) within the function “vegdist” in the Vegan Community Ecology Package.

## Supporting Information

Figure S1 **Bacterial community distribution in 17 ecosystems type.** A, the relative abundances of different phyla in 17 ecosystems type which including 125 sediments samples; B, the relative abundances of different classes in Proteobacteria of 17 ecosystem type. The relative abundance is presented in terms of percentage in total effective bacterial sequences in an ecosystem type. NH1, sample 19 in this study, NH2, the other 15 samples except sample 19 in this study, other ecosystem type were described in Table S1.
(TIF)

Table S1 **Datasets, location and parameters of marine sediments samples.**
(DOCX)
