# Correction: Bacterial Community Composition of South China Sea Sediments through Pyrosequencing-Based Analysis of 16S rRNA Genes

**DOI:** 10.1371/annotation/111eaadc-5af4-4d5e-8ae0-7abaebbb6ad6

**Published:** 2013-11-08

**Authors:** Daochen Zhu, Shoko-Hosoi Tanabe, Chong Yang, Weimin Zhang, Jianzhong Sun

A funding organization was incorrectly omitted from the Funding Statement. The Funding Statement should read: "This work was supported by a grant (Code: BK2012695) from the Natural Science Foundation of Jiangsu Province, China, by grants from the Priority Academic Program Development of Jiangsu Higher Education Institutions and by grants from the National High Technology Research and Development Program of China (863 Program, 2012AA092104), as well as by the Project Startup Foundation for Distinguished Scholars of Jiangsu University (Code: 10JDG084), the Chinese Academy of Sciences for Key Topics in Innovation Engineering (KSCX2-EW-G-12). Authors were also sponsored by the Scientific Research Foundation for the Returned Overseas Chinese Scholars, State Education Ministry. The funders had no role in study design, data collection and analysis, decision to publish, or preparation of the manuscript." 

