# Supplementary figures and images for: Bacterial Community Composition of South China Sea Sediments through Pyrosequencing-Based Analysis of 16S rRNA Genes
Source: PLoS One. 2013 Oct 21;8(10):e78501. doi: 10.1371/journal.pone.0078501 (PMC3804488; doi:10.1371/journal.pone.0078501)

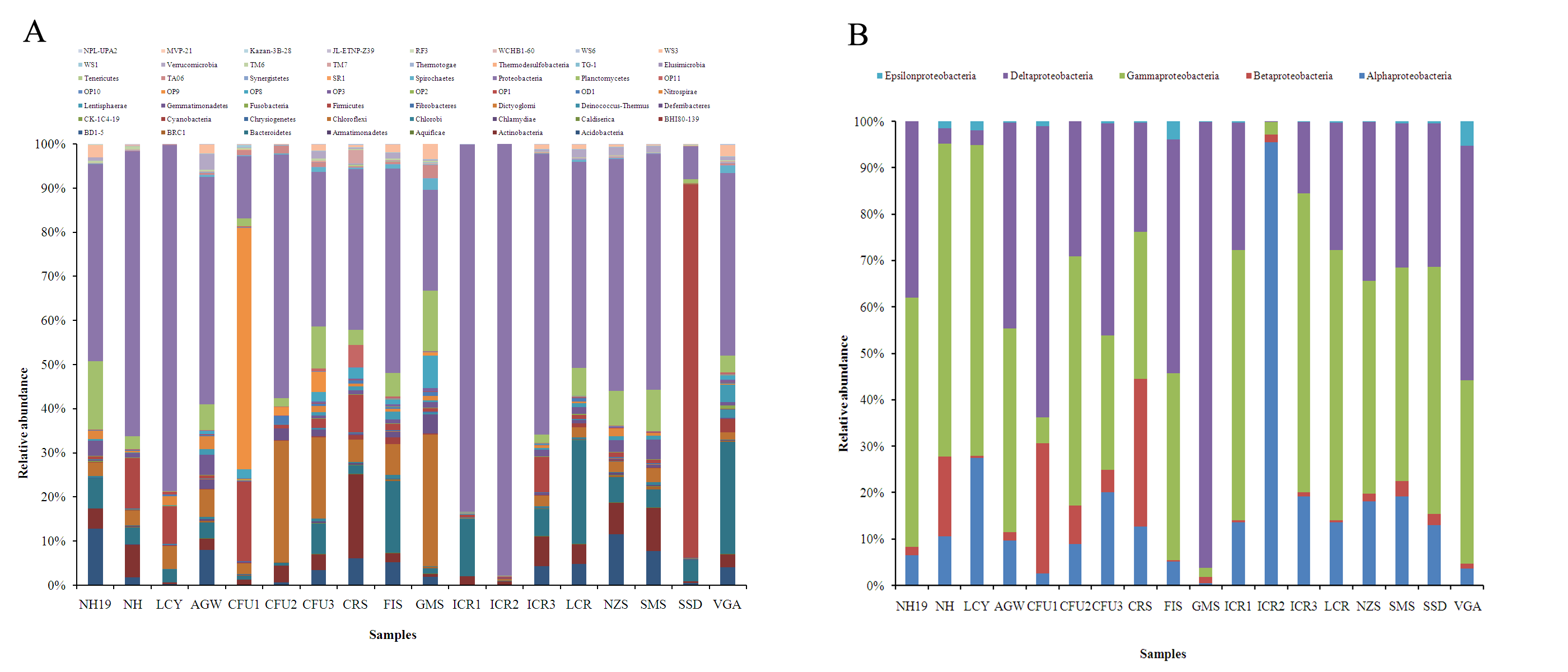

Supplement: Figure S1 — Bacterial community distribution in 17 ecosystems type. A, the relative abundances of different phyla in 17 ecosystems type which including 125 sediments samples; B, the relative abundances of different classes in Proteobacteria of 17 ecosystem type. The relative abundance is presented in terms of percentage in total effective bacterial sequences in an ecosystem type. NH1, sample 19 in this study, NH2, the other 15 samples except sample 19 in this study, other ecosystem type were described in Table S1. (TIF) [file pone.0078501.s001.tif]
