# Supplementary material for: Bacterial Community Composition of South China Sea Sediments through Pyrosequencing-Based Analysis of 16S rRNA Genes
Source: PLoS One. 2013 Oct 21;8(10):e78501. doi: 10.1371/journal.pone.0078501 (PMC3804488; doi:10.1371/journal.pone.0078501)
Supplement: Table S1 — Datasets, location and parameters of marine sediments samples. (DOCX) [file pone.0078501.s002.docx]

| Table S1. Datasets, location and parameters of marine sediments samples | | | | | | | | | |
| --- | --- | --- | --- | --- | --- | --- | --- | --- | --- |
| Symbol | Dataset | Sequence Archive accession # | Longitude (dd) | Latitude (dd) | Depth (m) | Date | Ecosystem type | Productivity Longhurst's index | Capture fisheries yield index |
| AGW | AGW_0001_2005_06_15 | SRA009839.1 | -53.18 | 5.54 | 1 | 15/06/2005 | Coastal | 5 | 4 |
|  | AGW_0002_2005_06_15 | SRA009839.1 | -53.18 | 5.54 | 1 | 15/06/2005 | Coastal | 5 | 4 |
|  | AGW_0003_2006_06_15 | SRA009839.1 | -52.2 | 5.54 | 52 | 15/06/2006 | Coastal | 5 | 4 |
|  | AGW_0004_2006_06_15 | SRA009839.1 | -52.2 | 5.54 | 52 | 15/06/2006 | Coastal | 5 | 4 |
| CFU1 | CFU_0001_2006_04_21 | SRA009850.1 | -9.51 | 35.56 | 3860 | 21/04/2006 | Open Ocean | 5 | 4 |
|  | CFU_0003_2006_04_24 | SRA009850.1 | -7.33 | 35.66 | 1326 | 24/04/2006 | Open Ocean | 5 | 4 |
|  | CFU_0005_2006_04_29 | SRA009850.1 | -7.33 | 35.66 | 1326 | 29/04/2006 | Open Ocean | 5 | 4 |
| CFU2 | CFU_0007_2002_03_03 | SRA009850.1 | -78.08 | -11.06 | 262 | 03/03/2002 | Open Ocean | 2 | 5 |
| CFU3 | CFU_0009_2002_06_01 | SRA009850.1 | 7.77 | 53.73 | 1 | 01/06/2002 | Coastal | 5 | 5 |
|  | CFU_0010_2002_06_01 | SRA009850.1 | 7.73 | 53.72 | 1 | 01/06/2002 | Coastal | 5 | 5 |
|  | CFU_0011_2006_10_25 | SRA009850.1 | 0.93 | 51.88 | 1 | 25/10/2006 | Coastal | 5 | 5 |
| CRS | CRS_0001_2008_11_07 | SRA009853.1 | -157.8 | 21.47 | 0.5 | 07/11/2008 | Coastal | 1 | 4 |
|  | CRS_0002_2008_11_07 | SRA009853.1 | -157.8 | 21.47 | 0.5 | 07/11/2008 | Coastal | 1 | 4 |
|  | CRS_0003_2008_11_07 | SRA009853.1 | -157.8 | 21.47 | 0.5 | 07/11/2008 | Coastal | 1 | 4 |
|  | CRS_0004_2008_11_07 | SRA009853.1 | -157.8 | 21.47 | 0.5 | 07/11/2008 | Coastal | 1 | 4 |
|  | CRS_0009_2008_11_07 | SRA009853.1 | -157.8 | 21.47 | 0.5 | 07/11/2008 | Coastal | 1 | 4 |
|  | CRS_0010_2008_11_07 | SRA009853.1 | -157.8 | 21.47 | 0.5 | 07/11/2008 | Coastal | 1 | 4 |
|  | CRS_0011_2008_11_07 | SRA009853.1 | -157.8 | 21.47 | 0.5 | 07/11/2008 | Coastal | 1 | 4 |
|  | CRS_0012_2008_11_07 | SRA009853.1 | -157.8 | 21.47 | 0.5 | 07/11/2008 | Coastal | 1 | 4 |
| FIS | FIS_0001_2008_04_10 | SRA009856.7 | 8.4 | 55.04 | 0.05 | 10/04/2008 | Coastal | 5 | 5 |
|  | FIS_0002_2008_04_10 | SRA009856.7 | 8.4 | 55.04 | 0.05 | 10/04/2008 | Coastal | 5 | 5 |
|  | FIS_0003_2008_04_10 | SRA009856.7 | 8.4 | 55.04 | 0.125 | 10/04/2008 | Coastal | 5 | 5 |
|  | FIS_0004_2008_04_10 | SRA009856.7 | 8.4 | 55.04 | 0.125 | 10/04/2008 | Coastal | 5 | 5 |
|  | FIS_0005_2008_04_11 | SRA009856.7 | 8.41 | 55.04 | 0.05 | 11/04/2008 | Coastal | 5 | 5 |
|  | FIS_0006_2008_04_11 | SRA009856.7 | 8.41 | 55.04 | 0.125 | 11/04/2008 | Coastal | 5 | 5 |
|  | FIS_0007_2008_04_11 | SRA009856.7 | 8.41 | 55.04 | 0.05 | 11/04/2008 | Coastal | 5 | 5 |
|  | FIS_0008_2008_04_11 | SRA009856.7 | 8.41 | 55.04 | 0.05 | 11/04/2008 | Coastal | 5 | 5 |
|  | FIS_0009_2008_04_11 | SRA009856.7 | 8.41 | 55.04 | 0.05 | 11/04/2008 | Coastal | 5 | 5 |
|  | FIS_0011_2008_04_11 | SRA009856.7 | 8.41 | 55.04 | 0.125 | 11/04/2008 | Coastal | 5 | 5 |
|  | FIS_0012_2008_04_11 | SRA009856.7 | 8.41 | 55.04 | 0.125 | 11/04/2008 | Coastal | 5 | 5 |
|  | FIS_0013_2008_04_12 | SRA009856.7 | 8.43 | 55.03 | 0.05 | 12/04/2008 | Coastal | 5 | 5 |
|  | FIS_0014_2008_04_12 | SRA009856.7 | 8.43 | 55.03 | 0.125 | 12/04/2008 | Coastal | 5 | 5 |
|  | GMS_0001_2006_12_04 | SRA009857.4 | -77.12 | 34.74 | 1 | 04/12/2006 | Coastal | 4 | 4 |
| ICR1 | ICR_0001_2007_05_17 | SRA009860.1 | 81.83 | 15.86 | 995 | 17/05/2007 | Coastal | 3 | 4 |
|  | ICR_0002_2007_05_17 | SRA009860.1 | 81.83 | 15.86 | 995 | 17/05/2007 | Coastal | 3 | 4 |
|  | ICR_0014_2007_05_17 | SRA009860.1 | 81.83 | 15.86 | 995 | 17/05/2007 | Coastal | 3 | 4 |
| ICR2 | ICR_0006_2005_04_15 | SRA009860.1 | 75.5 | -16 | 5000 | 15/04/2005 | Open Ocean | 1 | 4 |
| ICR3 | ICR_0003_2008_09_05 | SRA009860.1 | 73.88 | 15.51 | 0 | 05/09/2008 | Coastal | 3 | 4 |
|  | ICR_0004_2008_09_05 | SRA009860.1 | 73.8 | 15.65 | 0 | 05/09/2008 | Coastal | 3 | 4 |
|  | ICR_0007_2007_05_06 | SRA009860.1 | 73.29 | 17.06 | 0 | 06/05/2007 | Coastal | 3 | 4 |
|  | ICR_0008_2006_05_11 | SRA009860.1 | 73.28 | 17.06 | 0 | 11/05/2006 | Coastal | 3 | 4 |
|  | ICR_0011_2004_11_05 | SRA009860.1 | 73.29 | 17.06 | 0 | 05/11/2004 | Coastal | 3 | 4 |
|  | ICR_0012_2004_11_05 | SRA009860.1 | 73.29 | 17.06 | 0 | 05/11/2004 | Coastal | 3 | 4 |
|  | ICR_0015_2007_05_17 | SRA009860.1 | 73.9 | 15.12 | 0 | 17/05/2007 | Coastal | 3 | 4 |
|  | ICR_0016_2007_05_17 | SRA009860.1 | 73.9 | 15.12 | 0 | 17/05/2007 | Coastal | 3 | 4 |
| LCR | LCR_0001_2008_08_04 | SRA009862.3 | -67.05 | 17.94 | 2 | 04/08/2008 | Coastal | 2 | 4 |
|  | LCR_0003_2008_08_12 | SRA009862.3 | -67.04 | 17.88 | 50 | 12/08/2008 | Coastal | 2 | 4 |
|  | LCR_0015_2008_05_06 | SRA009862.3 | -64.12 | -42.43 | 0 | 06/05/2008 | Coastal | 4 | 4 |
|  | LCR_0016_2008_04_09 | SRA009862.3 | -67.37 | -45.75 | 0 | 09/04/2008 | Coastal | 4 | 4 |
| NZS | NZS_0001_2007_04_09 | SRA009906.1 | 179.63 | -43.98 | 529 | 09/04/2007 | Open Ocean | 3 | 3 |
|  | NZS_0002_2007_04_13 | SRA009906.1 | -175.55 | -43.29 | 644 | 13/04/2007 | Open Ocean | 3 | 3 |
|  | NZS_0003_2007_04_16 | SRA009906.1 | -176.71 | -42.78 | 1025 | 16/04/2007 | Open Ocean | 3 | 3 |
|  | NZS_0004_2007_04_26 | SRA009906.1 | 175.93 | -42.99 | 1197 | 26/04/2007 | Open Ocean | 3 | 3 |
|  | NZS_0005_2007_04_24 | SRA009906.1 | 178.99 | -42.99 | 530 | 24/04/2007 | Open Ocean | 3 | 3 |
|  | NZS_0006_2007_04_04 | SRA009906.1 | 178.64 | -44.13 | 516 | 04/04/2007 | Open Ocean | 3 | 3 |
|  | NZS_0007_2007_04_06 | SRA009906.1 | 177.14 | -44.49 | 1241 | 06/04/2007 | Open Ocean | 3 | 3 |
|  | NZS_0008_2007_04_05 | SRA009906.1 | 176.71 | -43.83 | 478 | 05/04/2007 | Open Ocean | 3 | 3 |
|  | NZS_0009_2007_04_17 | SRA009906.1 | -178.62 | -43.52 | 424 | 17/04/2007 | Open Ocean | 3 | 3 |
|  | NZS_0010_2007_04_07 | SRA009906.1 | 178.52 | -44.01 | 766 | 07/04/2007 | Open Ocean | 3 | 3 |
|  | NZS_0011_2007_04_20 | SRA009906.1 | -178.34 | -42.53 | 1400 | 20/04/2007 | Open Ocean | 3 | 3 |
|  | NZS_0012_2007_05_28 | SRA009906.1 | 167.53 | -38.62 | 482 | 28/05/2007 | Open Ocean | 2 | 3 |
|  | NZS_0013_2007_05_30 | SRA009906.1 | 167.53 | -36.92 | 1217 | 30/05/2007 | Open Ocean | 2 | 3 |
|  | NZS_0014_2007_06_07 | SRA009906.1 | 172.15 | -39.65 | 266 | 07/06/2007 | Open Ocean | 1 | 3 |
|  | NZS_0015_2007_06_05 | SRA009906.1 | 170.85 | -40.87 | 544 | 05/06/2007 | Open Ocean | 1 | 3 |
|  | NZS_0016_2007_06_04 | SRA009906.1 | 170.21 | -40.13 | 803 | 04/06/2007 | Open Ocean | 1 | 3 |
| SMS | SMS_0001_2007_09_19 | SRA009865.1 | -123.02 | 35.16 | 3953.5 | 19/09/2007 | Open Ocean | 3 | 4 |
|  | SMS_0002_2007_09_19 | SRA009865.1 | -123.02 | 35.16 | 3953.5 | 19/09/2007 | Open Ocean | 3 | 4 |
|  | SMS_0003_2007_09_19 | SRA009865.1 | -123.02 | 35.16 | 3953.5 | 19/09/2007 | Open Ocean | 3 | 4 |
|  | SMS_0004_2007_09_23 | SRA009865.1 | -123.02 | 35.16 | 3953.5 | 23/09/2007 | Open Ocean | 3 | 4 |
|  | SMS_0005_2007_09_23 | SRA009865.1 | -123.02 | 35.16 | 3953.5 | 23/09/2007 | Open Ocean | 3 | 4 |
|  | SMS_0006_2007_09_23 | SRA009865.1 | -123.02 | 35.16 | 3953.5 | 23/09/2007 | Open Ocean | 3 | 4 |
|  | SMS_0007_2007_09_19 | SRA009865.1 | -123.02 | 35.16 | 3953.5 | 19/09/2007 | Open Ocean | 3 | 4 |
|  | SMS_0008_2007_09_19 | SRA009865.1 | -123.02 | 35.16 | 3953.5 | 19/09/2007 | Open Ocean | 3 | 4 |
|  | SMS_0009_2007_09_19 | SRA009865.1 | -123.02 | 35.16 | 3953.5 | 19/09/2007 | Open Ocean | 3 | 4 |
|  | SMS_0010_2007_09_23 | SRA009865.1 | -123.02 | 35.16 | 3953.5 | 23/09/2007 | Open Ocean | 3 | 4 |
|  | SMS_0011_2007_09_23 | SRA009865.1 | -123.02 | 35.16 | 3953.5 | 23/09/2007 | Open Ocean | 3 | 4 |
|  | SMS_0012_2007_09_23 | SRA009865.1 | -123.02 | 35.16 | 3953.5 | 23/09/2007 | Open Ocean | 3 | 4 |
|  | SMS_0013_2007_09_19 | SRA009865.1 | -123.02 | 35.16 | 3953.5 | 19/09/2007 | Open Ocean | 3 | 4 |
|  | SMS_0014_2007_09_19 | SRA009865.1 | -123.02 | 35.15 | 3953.5 | 19/09/2007 | Open Ocean | 3 | 4 |
|  | SMS_0015_2007_09_23 | SRA009865.1 | -123.02 | 35.16 | 3953.5 | 23/09/2007 | Open Ocean | 3 | 4 |
|  | SMS_0016_2007_09_23 | SRA009865.1 | -123.02 | 35.16 | 3953.5 | 23/09/2007 | Open Ocean | 3 | 4 |
| SSD | SSD_0001_2008_12_03 | SRA009867.1 | -70 | 42 | 0.01 | 03/12/2008 | Coastal | 4 | 4 |
|  | SSD_0002_2008_12_03 | SRA009867.1 | -70 | 42 | 0.01 | 03/12/2008 | Coastal | 4 | 4 |
|  | SSD_0003_2008_12_03 | SRA009867.1 | -70 | 42 | 0.01 | 03/12/2008 | Coastal | 4 | 4 |
|  | SSD_0004_2008_12_03 | SRA009867.1 | -70 | 42 | 0.01 | 03/12/2008 | Coastal | 4 | 4 |
|  | SSD_0005_2008_12_03 | SRA009867.1 | -70 | 42 | 0.01 | 03/12/2008 | Coastal | 4 | 4 |
|  | SSD_0006_2008_12_03 | SRA009867.1 | -70 | 42 | 0.01 | 03/12/2008 | Coastal | 4 | 4 |
|  | SSD_0007_2008_12_03 | SRA009867.1 | -70 | 42 | 0.01 | 03/12/2008 | Coastal | 4 | 4 |
|  | SSD_0008_2008_12_03 | SRA009867.1 | -70 | 42 | 0.01 | 03/12/2008 | Coastal | 4 | 4 |
| VAG | VAG_0001_2007_12_20 | SRA009868.2 | -73.07 | -36.69 | 15 | 20/12/2007 | Coastal | 2 | 5 |
|  | VAG_0002_2007_12_20 | SRA009868.2 | -73.04 | -36.64 | 27 | 20/12/2007 | Coastal | 2 | 5 |
|  | VAG_0003_2007_12_20 | SRA009868.2 | -73 | -36.6 | 35 | 20/12/2007 | Coastal | 2 | 5 |
|  | VAG_0004_2008_01_08 | SRA009868.2 | -73 | -36.6 | 88 | 08/01/2008 | Coastal | 2 | 5 |
|  | VAG_0005_2008_04_01 | SRA009868.2 | -73.07 | -36.69 | 15 | 01/04/2008 | Coastal | 2 | 5 |
|  | VAG_0006_2008_04_01 | SRA009868.2 | -73.04 | -36.64 | 27 | 01/04/2008 | Coastal | 2 | 5 |
|  | VAG_0007_2008_04_01 | SRA009868.2 | -73 | -36.6 | 35 | 01/04/2008 | Coastal | 2 | 5 |
|  | VAG_0008_2008_04_21 | SRA009868.2 | -73.12 | -36.51 | 88 | 21/04/2008 | Coastal | 2 | 5 |
|  | VAG_0009_2008_09_29 | SRA009868.2 | -73.07 | -36.69 | 15 | 29/09/2008 | Coastal | 2 | 5 |
|  | VAG_0010_2008_09_29 | SRA009868.2 | -73.04 | -36.64 | 27 | 29/09/2008 | Coastal | 2 | 5 |
|  | VAG_0011_2008_09_29 | SRA009868.2 | -73 | -36.6 | 35 | 29/09/2008 | Coastal | 2 | 5 |
|  | VAG_0012_2008_09_23 | SRA009868.2 | -73.12 | -36.51 | 88 | 23/09/2008 | Coastal | 2 | 5 |
|  | VAG_0013_2009_01_20 | SRA009868.2 | -73.07 | -36.69 | 15 | 20/01/2009 | Coastal | 2 | 5 |
|  | VAG_0014_2009_01_20 | SRA009868.2 | -73.04 | -36.64 | 27 | 20/01/2009 | Coastal | 2 | 5 |
|  | VAG_0015_2009_01_20 | SRA009868.2 | -73 | -36.6 | 35 | 20/01/2009 | Coastal | 2 | 5 |
|  | VAG_0016_2009_01_15 | SRA009868.2 | -73.12 | -36.51 | 88 | 15/01/2009 | Coastal | 2 | 5 |
